# Supplementary material for: Effect of Physical Activity on Metabolic Syndrome Markers in Adults with Type 2 Diabetes: A Systematic Review and Meta-Analysis
Source: Sports (Basel). 2023 May 9;11(5):101. doi: 10.3390/sports11050101 (PMC10222387; doi:10.3390/sports11050101)
Supplement: Supplementary file 1 [file sports-11-00101-s001.zip › sports-2307117-supplementary.pdf]

S1: Search Strategy

MEDLINE Complete (via EBSCOhost platform)

S63 S19 AND S33 AND S54 AND S61 Limiters - English Language	**569**
S62 S19 AND S33 AND S54 AND S61	583
S61 S55 OR S56 OR S57 OR S58 OR S59 OR S60	307,434
S60 MH "Randomized Controlled Trials as Topic+")	143,468
S59 TI RCT OR AB RCT	51,283
S58 TI "equivalence trial*" OR AB "equivalence trial*"	470
S57 TI "pragmatic clinical trial*" OR AB "pragmatic clinical trial*"	473
S56 TI "randomised control* trial*" OR AB "randomised control* trial*"	47,660
S55 TI "randomized control* trial*" OR AB "randomized control* trial*"	151,528
S54 S34 OR S35 OR S36 OR S37 OR S38 OR S39 OR S40 OR S41 OR S42 OR S43 OR S44 OR S45 OR S46 OR S47 OR S48 OR S49 OR S50 OR S51 OR S52 OR S53 OR S54 OR S55	668,495
S53 MH "Metabolic Syndrome")	32,515
S52 TI "waist-to-hip ratio" OR AB "waist-to-hip ratio"	5,867
S51 TI "waist circumference" OR AB "waist circumference"	28,079
S50 TI "glycosylated haemoglobin" OR AB "glycosylated haemoglobin"	2,442
S49 TI "glycosylated hemoglobin" OR AB "glycosylated hemoglobin"	7,677
S48 TI "glycated hemoglobin" OR AB "glycated hemoglobin"	8,702
S47 TI "glycated haemoglobin" OR AB "glycated haemoglobin"	3,722
S46 TI "low-density lipoprotein" OR AB "low-density lipoprotein"	68,126
S45 TI "high-density lipoprotein" OR AB "high-density lipoprotein"	50,204
S44 TI "high-density lipoprotein ratio" OR AB "high-density lipoprotein ratio"	304
S43 TI cholesterol OR AB cholesterol	244,707
S42 TI triglyceride* OR AB triglyceride*	114,461
S41 TI "blood pressure" OR AB "blood pressure"	299,406
S40 TI "cardiovascular syndrome" OR AB "cardiovascular syndrome"	155
S39 TI "metabolic cardiovascular" OR AB "metabolic cardiovascular"	625
S38 TI "cardiovascular risk factor*" OR AB "cardiovascular risk factor*"	35,464
S37 TI cardiometabolic OR AB cardiometabolic	12,480
S36 TI "syndrome X" OR AB "syndrome X"	1,778
S35 TI "metabolic X" OR AB "metabolic X"	29
S34 TI "metabolic syndrome" OR AB "metabolic syndrome"	52,162

S33 S20 OR S21 OR S22 OR S23 OR S24 OR S25 OR S26 OR S27 OR S28 OR S29 OR S30 OR S31 OR S32	201,169
S32 (MH "Diabetes Mellitus, Type 2+")	137,449
S31 TI “stable diabet*” OR AB “stable diabet*”	151
S30 TI “late-onset diabet*” OR AB “late-onset diabet*”	120
S29 TI “adult* diabet*” OR AB “adult* diabet*”	1,269
S28 TI “non-insulin depend*” OR AB “non-insulin depend*”	10,941
S27 TI “slow onset diabet*” OR AB “slow onset diabet*”	4
S26 TI “matur* onset diabet*” OR AB “matur* onset diabet*”	2,073
S25 TI DM2 OR AB DM2	2,009
S24 TI T2DM OR AB T2DM	22,256
S23 TI T2D OR AB T2D	10,798
S22 TI NIDDM OR AB NIDDM	6,953
S21 TI “type II diabet*” OR AB “type II diabet*”	9,958
S20 TI "type 2 diabet*" OR AB “type 2 diabet*”	136,731
S19 S1 OR S2 OR S3 OR S4 OR S5 OR S6 OR S7 OR S8 OR S9 OR S10 OR S11 OR S12 OR S13 OR S14 OR S15 OR S16 OR S17 OR S18	787,343
S18 (MH "Exercise+")	202,972
S17 TI “endur* train*” OR AB “endur* train*”	6,137
S16 TI "active transport*” OR AB "active transport*”	8,637
S15 TI bicycl* OR AB bicycl*	24,303
S14 TI cycling OR AB cycling	63,451
S13 TI “strength train*” OR AB “strength train*”	5,279
S12 TI “cardio fitness” OR AB “cardio fitness”	9
S11 TI “cardio train*” OR AB “cardio train*”	9
S10 TI jogging OR AB jogging	1,681
S9 TI running OR AB running	61,085
S8 TI pilate* OR AB pilate*	585
S7 TI yoga OR AB yoga	5,044
S6 TI aerobic* OR AB aerobic*	90,346
S5 TI "resistance train*" OR AB “resistance train*”	8,752
S4 TI Sport* OR AB Sport*	78,382
S3 TI walk* OR AB walk*	121,817
S2 TI exercis* OR AB exercis*	302,741
S1 TI “Physical* Activ*” OR AB “Physical* Activ*”	121,599

Limiters: English,
Explanatory notes: All keywords are searched using Title and Abstract. TI – Title search. AB – Abstract search. MH – Exact Subject heading search. The * at the end of a MH term means this term has been exploded

CINAHL (via EBSCOhost platform)

S63	S19 AND S33 AND S54 AND S61 Limiters - English Language	**393**
S62	S19 AND S33 AND S54 AND S61	400
S61	S57 OR S58 OR S59 OR S60	186,992
S60	(MH "Randomized Controlled Trials+")	113,253
S59	TI RCT OR AB RCT	22,155
S58	TI “equivalence trial*” OR AB “equivalence trial*”	191
S57	TI “pragmatic clinical trial*” OR AB “pragmatic clinical trial*”	243
S56	TI “randomised control* trial*” OR AB “randomised control* trial*”	25,410
S55	TI “randomized control* trial*” OR AB “randomized control* trial*”	77,573
S54	S34 OR S35 OR S36 OR S37 OR S38 OR S39 OR S40 OR S41 OR S42 OR S43 OR S44 OR S45 OR S46 OR S47 OR S48 OR S49 OR S50 OR S51 OR S52 OR S53 OR S54 OR S55	142,100
S53	(MH "Metabolic Syndrome X+")	13,675
S52	TI “waist-to-hip ratio” OR AB “waist-to-hip ratio”	1,793
S51	TI “waist circumference” OR AB “waist circumference”	10,511
S50	TI "glycosylated haemoglobin" OR AB “glycosylated haemoglobin”	586
S49	TI “glycosylated hemoglobin” OR AB “glycosylated hemoglobin”	2,368
S48	TI “glycated hemoglobin” OR AB “glycated hemoglobin”	2,978
S47	TI “glycated haemoglobin” OR AB “glycated haemoglobin”	999
S46	TI “low-density lipoprotein” OR AB “low-density lipoprotein”	12,322
S45	TI “high-density lipoprotein” OR AB “high-density lipoprotein"	10,575
S44	TI “high-density lipoprotein ratio” OR AB “high-density lipoprotein ratio”	93
S43	TI cholesterol OR AB cholesterol	39,758
S42	TI triglyceride* OR AB triglyceride*	18,864
S41	TI “blood pressure” OR AB “blood pressure”	70,243
S40	TI “cardiovascular syndrome” OR AB “cardiovascular syndrome”	18
S39	TI “metabolic cardiovascular” OR AB “metabolic cardiovascular"	113

S38 TI “cardiovascular risk factor*” OR AB “cardiovascular risk factor*”	10,610
S37 TI cardiometabolic OR AB cardiometabolic	5,497
S36 TI “syndrome X” OR AB “syndrome X”	352
S35 TI “metabolic X” OR AB “metabolic X”	3
S34 TI “metabolic syndrome” OR AB “metabolic syndrome”	15,198
S33 S20 OR S21 OR S22 OR S23 OR S24 OR S25 OR S26 OR S27 OR S28 OR S29 OR S30 OR S31 OR S32	79,660
S32 (MH "Diabetes Mellitus, Type 2")	63,135
S31 TI “stable diabet*” OR AB “stable diabet*”	23
S30 TI “late-onset diabet*” OR AB “late-onset diabet*”	21
S29 TI “adult* diabet*” OR AB “adult* diabet*”	437
S28 TI “non-insulin depend*” OR AB “non-insulin depend*”	1,420
S27 TI “slow onset diabet*” OR AB “slow onset diabet*”	2
S26 TI “matur* onset diabet*” OR AB “matur* onset diabet*”	454
S25 TI DM2 OR AB DM2	449
S24 TI T2DM OR AB T2DM	6,210
S23 TI T2D OR AB T2D	3,293
S22 TI NIDDM OR AB NIDDM	1,315
S21 TI “type II diabet*” OR AB “type II diabet*”	1,948
S20 TI "type 2 diabet*" OR AB “type 2 diabet*”	52,110
S19 S1 OR S2 OR S3 OR S4 OR S5 OR S6 OR S7 OR S8 OR S9 OR S10 OR S11 OR S12 OR S13 OR S14 OR S15 OR S16 OR S17 OR S18	290,206
S18 (MH "Physical Activity")	42,894
S17 TI “endur* train*” OR AB “endur* train*”	1,932
S16 TI "active transport*” OR AB "active transport*”	532
S15 TI bicycl* OR AB bicycl*	3,479
S14 TI cycling OR AB cycling	7,938
S13 TI “strength train*” OR AB “strength train*”	3,437
S12 TI “cardio fitness” OR AB “cardio fitness”	8
S11 TI “cardio train*” OR AB “cardio train*”	9
S10 TI jogging OR AB jogging	660
S9 TI running OR AB running	15,986
S8 TI pilate* OR AB pilate*	1,066
S7 TI yoga OR AB yoga	7,325
S6 TI aerobic* OR AB aerobic*	14,349

S5	TI "resistance train*" OR AB “resistance train*”	5,167
S4	TI Sport* OR AB Sport*	48,940
S3	TI walk* OR AB walk*	47,404
S2	TI exercis* OR AB exercis*	123,607
S1	TI “Physical* Activ*” OR AB “Physical* Activ*”	64,858

Limiters: English,

Explanatory notes: All keywords are searched using Title and Abstract. TI – Title search. AB – Abstract search. MH – Exact Subject heading search. The * at the end of a MH term means this term has been exploded

PSYCHINFO (Via EBSCOhost platform)

S63	S19 AND S33 AND S54 AND S62 Limiters – English	39
S62	S19 AND S33 AND S54 AND S62	39
S61	S55 OR S56 OR S57 OR S58 OR S59 OR S60	38,068
S60	DE "Randomized Controlled Trials" OR DE "Randomized Clinical Trials"	851
S59	TI RCT OR AB RCT	8,245
S58	TI “equivalence trial*” OR AB “equivalence trial*”	59
S57	TI “pragmatic clinical trial*” OR AB “pragmatic clinical trial*”	62
S56	TI “randomised control* trial*” OR AB “randomised control* trial*”	5,965
S55	TI “randomized control* trial*” OR AB “randomized control* trial*”	29,301
S54	S34 OR S35 OR S36 OR S37 OR S38 OR S39 OR S40 OR S41 OR S42 OR S43 OR S44 OR S45 OR S46 OR S47 OR S48 OR S49 OR S50 OR S51 OR S52 OR S53 OR S54 OR S55	34,205
S53	DE "Metabolic Syndrome"	2,322
S52	TI “waist-to-hip ratio” OR AB “waist-to-hip ratio”	550
S51	TI “waist circumference” OR AB “waist circumference”	2,710
S50	TI "glycosylated haemoglobin" OR AB “glycosylated haemoglobin”	81
S49	TI “glycosylated hemoglobin” OR AB “glycosylated hemoglobin”	545
S48	TI “glycated hemoglobin” OR AB “glycated hemoglobin”	358
S47	TI “glycated haemoglobin” OR AB “glycated haemoglobin”	103
S46	TI “low-density lipoprotein” OR AB “low-density lipoprotein”	1,715
S45	TI “high-density lipoprotein” OR AB “high-density lipoprotein"	1,694
S44	TI “high-density lipoprotein ratio” OR AB “high-density lipoprotein ratio”	17
S43	TI cholesterol OR AB cholesterol	8,234
S42	TI triglyceride* OR AB triglyceride*	3,157
S41	TI “blood pressure” OR AB “blood pressure”	19,666

S40	TI “cardiovascular syndrome” OR AB “cardiovascular syndrome”	5
S39	TI “metabolic cardiovascular” OR AB “metabolic cardiovascular"	64
S38	TI “cardiovascular risk factor*” OR AB “cardiovascular risk factor*”	2,279
S37	TI cardiometabolic OR AB cardiometabolic	1,116
S36	TI “syndrome X” OR AB “syndrome X”	61
S35	TI “metabolic X” OR AB “metabolic X”	1
S34	TI “metabolic syndrome” OR AB “metabolic syndrome”	3,396
S33	S20 OR S21 OR S22 OR S23 OR S24 OR S25 OR S26 OR S27 OR S28 OR S29 OR S30 OR S31 OR S32	9,368
S32	DE "Type 2 Diabetes" OR DE "Blood Sugar"	4,773
S31	TI “stable diabet*” OR AB “stable diabet*”	5
S30	TI “late-onset diabet*” OR AB “late-onset diabet*”	3
S29	TI “adult* diabet*” OR AB “adult* diabet*”	99
S28	TI “non-insulin depend*” OR AB “non-insulin depend*”	207
S27	TI “slow onset diabet*” OR AB “slow onset diabet*”	0
S26	TI “matur* onset diabet*” OR AB “matur* onset diabet*”	11
S25	TI DM2 OR AB DM2	143
S24	TI T2DM OR AB T2DM	1,057
S23	TI T2D OR AB T2D	480
S22	TI NIDDM OR AB NIDDM	96
S21	TI “type II diabet*” OR AB “type II diabet*”	687
S20	TI "type 2 diabet*" OR AB “type 2 diabet*”	7,151
S19	S1 OR S2 OR S3 OR S4 OR S5 OR S6 OR S7 OR S8 OR S9 OR S10 OR S11 OR S12 OR S13 OR S14 OR S15 OR S16 OR S17 OR S18	174,149
S18	DE "Physical Activity" OR DE "Actigraphy" OR DE "Exercise"	45,176
S17	TI “endur* train*” OR AB “endur* train*”	288
S16	TI "active transport*” OR AB "active transport*”	447
S15	TI bicycl* OR AB bicycl*	2,321
S14	TI cycling OR AB cycling	5,192
S13	TI “strength train*” OR AB “strength train*”	616
S12	TI “cardio fitness” OR AB “cardio fitness”	1
S11	TI “cardio train*” OR AB “cardio train*”	3
S10	TI jogging OR AB jogging	318
S9	TI running OR AB running	15,994
S8	TI pilate* OR AB pilate*	103

S7	TI yoga OR AB yoga	2,913
S6	TI aerobic* OR AB aerobic*	4,786
S5	TI "resistance train*" OR AB “resistance train*”	749
S4	TI Sport* OR AB Sport*	35,076
S3	TI walk* OR AB walk*	27,090
S2	TI exercis* OR AB exercis*	68,383
S1	TI “Physical* Activ*” OR AB “Physical* Activ*”	36,492

Limiters: English,

Explanatory notes: All keywords are searched using Title and Abstract. TI – Title search. AB – Abstract search. MH – Exact Subject heading search. The * at the end of a MH term means this term has been exploded

SPORTSDISCUS (via EBSCOhost platform)

S63	S19 AND S33 AND S56 AND S62	33
S62	S19 AND S33 AND S56 AND S62	33
S61	S57 OR S58 OR S59 OR S60 OR S61	12,324
S60	TI RCT OR AB RCT	1,985
S59	TI “equivalence trial*” OR AB “equivalence trial*”	10
S58	TI “pragmatic clinical trial*” OR AB “pragmatic clinical trial*”	17
S57	TI “randomised control* trial*” OR AB “randomised control* trial*”	2,048
S56	TI “randomized control* trial*” OR AB “randomized control* trial*”	9,702
S55	S34 OR S35 OR S36 OR S37 OR S38 OR S39 OR S40 OR S41 OR S42 OR S43 OR S44 OR S45 OR S46 OR S47 OR S48 OR S49 OR S50 OR S51 OR S52 OR S53 OR S54 OR S55	43,918
S54	DE "Metabolic Syndrome"	1,557
S53	TI “waist-to-hip ratio” OR AB “waist-to-hip ratio”	411
S51	TI “waist circumference” OR AB “waist circumference”	2,467
S50	TI "glycosylated haemoglobin" OR AB “glycosylated haemoglobin”	136
S49	TI “glycosylated hemoglobin” OR AB “glycosylated hemoglobin”	143
S48	TI “glycated hemoglobin” OR AB “glycated hemoglobin”	182
S47	TI “glycated haemoglobin” OR AB “glycated haemoglobin”	62
S46	TI “low-density lipoprotein” OR AB “low-density lipoprotein”	1,738
S45	TI “high-density lipoprotein” OR AB “high-density lipoprotein"	1,868
S44	TI “high-density lipoprotein ratio” OR AB “high-density lipoprotein ratio”	10

S43	TI cholesterol OR AB cholesterol	7,722
S42	TI triglyceride* OR AB triglyceride*	4,036
S41	TI “blood pressure” OR AB “blood pressure”	16,427
S40	TI “cardiovascular syndrome” OR AB “cardiovascular syndrome”	3
S39	TI “metabolic cardiovascular” OR AB “metabolic cardiovascular"	43
S38	TI “cardiovascular risk factor*” OR AB “cardiovascular risk factor*”	1,293
S37	TI cardiometabolic OR AB cardiometabolic	1,103
S36	TI “syndrome X” OR AB “syndrome X”	41
S35	TI “metabolic X” OR AB “metabolic X”	1
S34	TI “metabolic syndrome” OR AB “metabolic syndrome”	2,574
S33	S20 OR S21 OR S22 OR S23 OR S24 OR S25 OR S26 OR S27 OR S28 OR S29 OR S30 OR S31 OR S32	5,465
S32	DE "TYPE 2 diabetes"	2,223
S31	TI “stable diabet*” OR AB “stable diabet*”	1
S30	TI “late-onset diabet*” OR AB “late-onset diabet*”	0
S29	TI “adult* diabet*” OR AB “adult* diabet*”	25
S28	TI “non-insulin depend*” OR AB “non-insulin depend*”	194
S27	TI “slow onset diabet*” OR AB “slow onset diabet*”	0
S26	TI “matur* onset diabet*” OR AB “matur* onset diabet*”	13
S25	TI DM2 OR AB DM2	46
S24	TI T2DM OR AB T2DM	485
S23	TI T2D OR AB T2D	341
S22	TI NIDDM OR AB NIDDM	78
S21	TI “type II diabet*” OR AB “type II diabet*”	333
S20	TI "type 2 diabet*" OR AB “type 2 diabet*”	4,580
S19	S1 OR S2 OR S3 OR S4 OR S5 OR S6 OR S7 OR S8 OR S9 OR S10 OR S11 OR S12 OR S13 OR S14 OR S15 OR S16 OR S17 OR S18	604,438
S18	DE "PHYSICAL activity"	19,693
S17	TI “endur* train*” OR AB “endur* train*”	4,590
S16	TI "active transport*” OR AB "active transport*”	405
S15	TI bicycl* OR AB bicycl*	24,141
S14	TI cycling OR AB cycling	29,354
S13	TI “strength train*” OR AB “strength train*”	8,600
S12	TI “cardio fitness” OR AB “cardio fitness”	44
S11	TI “cardio train*” OR AB “cardio train*”	69

S10	TI jogging OR AB jogging	2,081
S9	TI running OR AB running	53,282
S8	TI pilate* OR AB pilate*	1,763
S7	TI yoga OR AB yoga	9,482
S6	TI aerobic* OR AB aerobic*	21,248
S5	TI "resistance train*" OR AB “resistance train*”	7,547
S4	TI Sport* OR AB Sport*	323,696
S3	TI walk* OR AB walk*	34,990
S2	TI exercis* OR AB exercis*	143,863
S1	TI “Physical* Activ*” OR AB “Physical* Activ*”	49,673

Limiters: English,

Explanatory notes: All keywords are searched using Title and Abstract. TI – Title search. AB – Abstract search. MH – Exact Subject heading search. The * at the end of a MH term means this term has been exploded

SOCINDEX (via EBSCOhost platform)

S62	S19 AND S33 AND S53 AND S60	**5**
S61	S19 AND S33 AND S53 AND S60	5
S60	S54 OR S55 OR S56 OR S57 OR S58 OR S59	5,679
S59	DE "RANDOMIZED controlled trials"	2,573
S58	TI RCT OR AB RCT	799
S57	TI “equivalence trial*” OR AB “equivalence trial*”	3
S56	TI “pragmatic clinical trial*” OR AB “pragmatic clinical trial*”	20
S55	TI “randomised control* trial*” OR AB “randomised control* trial*”	636
S54	TI “randomized control* trial*” OR AB “randomized control* trial*”	3,456
S53	S34 OR S35 OR S36 OR S37 OR S38 OR S39 OR S40 OR S41 OR S42 OR S43 OR S44 OR S45 OR S46 OR S47 OR S48 OR S49 OR S50 OR S51 OR S52 OR S53 OR S54	4,254
S52	TI “waist-to-hip ratio” OR AB “waist-to-hip ratio”	90
S51	TI “waist circumference” OR AB “waist circumference”	294
S50	TI "glycosylated haemoglobin" OR AB “glycosylated haemoglobin”	12
S49	TI “glycosylated hemoglobin” OR AB “glycosylated hemoglobin”	114
S48	TI “glycated hemoglobin” OR AB “glycated hemoglobin”	34
S47	TI “glycated haemoglobin” OR AB “glycated haemoglobin”	2

S46	TI “low-density lipoprotein” OR AB “low-density lipoprotein”	193
S45	TI “high-density lipoprotein” OR AB “high-density lipoprotein"	262
S44	TI “high-density lipoprotein ratio” OR AB “high-density lipoprotein ratio”	1
S43	TI cholesterol OR AB cholesterol	1,32
S42	TI triglyceride* OR AB triglyceride*	30
S41	TI “blood pressure” OR AB “blood pressure”	2,517
S40	TI “cardiovascular syndrome” OR AB “cardiovascular syndrome”	362
S39	TI “metabolic cardiovascular” OR AB “metabolic cardiovascular"	5
S38	TI “cardiovascular risk factor*” OR AB “cardiovascular risk factor*”	383
S37	TI cardiometabolic OR AB cardiometabolic	86
S36	TI “syndrome X” OR AB “syndrome X”	10
S35	TI “metabolic X” OR AB “metabolic X”	41
S34	TI “metabolic syndrome” OR AB “metabolic syndrome”	243
S33	S20 OR S21 OR S22 OR S23 OR S24 OR S25 OR S26 OR S27 OR S28 OR S29 OR S30 OR S31 OR S32	2,296
S32	DE "DIABETES"	1,613
S31	TI “stable diabet*” OR AB “stable diabet*”	0
S30	TI “late-onset diabet*” OR AB “late-onset diabet*”	1
S29	TI “adult* diabet*” OR AB “adult* diabet*”	12
S28	TI “non-insulin depend*” OR AB “non-insulin depend*”	70
S27	TI “slow onset diabet*” OR AB “slow onset diabet*”	0
S26	TI “matur* onset diabet*” OR AB “matur* onset diabet*”	5
S25	TI DM2 OR AB DM2	5
S24	TI T2DM OR AB T2DM	44
S23	TI T2D OR AB T2D	18
S22	TI NIDDM OR AB NIDDM	41
S21	TI “type II diabet*” OR AB “type II diabet*”	69
S20	TI "type 2 diabet*" OR AB “type 2 diabet*”	825
S19	S1 OR S2 OR S3 OR S4 OR S5 OR S6 OR S7 OR S8 OR S9 OR S10 OR S11 OR S12 OR S13 OR S14 OR S15 OR S16 OR S17 OR S18	59,912
S18	DE "PHYSICAL fitness" OR DE "SPORTS"	5,860
S17	TI “endur* train*” OR AB “endur* train*”	12

S16	TI "active transport*" OR AB "active transport*"	52
S15	TI bicycl* OR AB bicycl*	958
S14	TI cycling OR AB cycling	595
S13	TI “strength train*” OR AB “strength train*”	81
S12	TI “cardio fitness” OR AB “cardio fitness”	9
S11	TI “cardio train*” OR AB “cardio train*”	1
S10	TI jogging OR AB jogging	86
S9	TI running OR AB running	5,240
S8	TI pilate* OR AB pilate*	29
S7	TI yoga OR AB yoga	423
S6	TI aerobic* OR AB aerobic*	432
S5	TI "resistance train*" OR AB “resistance train*”	103
S4	TI Sport* OR AB Sport*	17,267
S3	TI walk* OR AB walk*	8,472
S2	TI exercis* OR AB exercis*	24,194
S1	TI “Physical* Activ*” OR AB “Physical* Activ*”	5,428

Limiters: English,

Explanatory notes: All keywords are searched using Title and Abstract. TI – Title search. AB – Abstract search. MH – Exact Subject heading search. The * at the end of a MH term means this term has been exploded

EMBASE SEARCH (Embase Database on the Embase platform)

#63 #19 AND #33 AND #54 AND #61 AND [humans]/lim AND [english]/lim AND [embase]/lim		**1,148**
#62	#19 AND #33 AND #54 AND #61	1,432
#61	#55 OR #56 OR #57 OR #58 OR #59 OR #60	785,271
#60	'randomized controlled trial'/exp	641569
#59	'equivalence trial*':ab,ti	593
#58	rct:ab,ti	40659
#57	'pragmatic clinical trial*':ab,ti	612
#56	'randomised control* trial*':ab,ti	62894
#55	'randomized control* trial*':ab,ti	197737

#54	#34 OR #35 OR #36 OR #37 OR #38 OR #39 OR #40 OR #41 OR #42 OR #43 OR #44 OR #45 OR #46 OR #47 OR #48 OR #49 OR #50 OR #51 OR #52 OR #53	948,377
#53	'metabolic syndrome x'/exp	85889
#52	'waist-to-hip ratio':ab,ti	7983
#51	'waist circumference':ab,ti	45299
#50	'glycosylated haemoglobin':ab,ti	3281
#49	'glycosylated hemoglobin':ab,ti	10319
#48	'glycated hemoglobin':ab,ti	11843
#47	'glycated haemoglobin':ab,ti	5005
#46	'low-density lipoprotein':ab,ti	83106
#45	'high-density lipoprotein':ab,ti	61718
#44	'high-density lipoprotein ratio':ab,ti	6364
#43	cholesterol:ab,ti	329794
#42	triglyceride*:ab,ti	164488
#41	'blood pressure':ab,ti	432545
#40	'cardiovascular syndrome':ab,ti	184
#39	'metabolic cardiovascular':ab,ti	902
#38	'cardiovascular risk factor*':ab,ti	56048
#37	cardiometabolic:ab,ti	20597
#36	'syndrome x':ab,ti	2339
#35	'metabolic x':ab,ti	41
#34	'metabolic syndrome':ab,ti	81889
#33	#20 OR #21 OR #22 OR #23 OR #25 OR #26 OR #27 OR #28 OR #29 OR #30 OR #31 OR #32	323468
#32	'non insulin dependent diabetes mellitus'/exp	270632
#31	'stable diabet*':ab,ti	222
#30	'late-onset diabet*':ab,ti	158
#29	'adult* diabet*':ab,ti	1939

#28	'non-insulin depend*':ab,ti	12718
#27	'slow onset diabet*':ab,ti	5
#26	'matur* onset diabet*':ab,ti	2936
#25	dm2:ab,ti	4524
#24	t2dm:ab,ti	39782
#23	t2d:ab,ti	20958
#22	niddm:ab,ti	802
#21	'type ii diabet*':ab,ti	15538
#20	'type 2 diabet*':ab,ti	211853
#19	#1 OR #2 OR #3 OR #4 OR #5 OR #6 OR #7 OR #8 OR #9 OR #10 OR #11 OR #12 OR #13 OR #14 OR #15 OR #16 OR #17 OR #18	1171518
#18	'physical activity'/exp	442340
#17	'endur* train*':ab,ti	7753
#16	'active transport*':ab,ti	9667
#15	bicycl*:ab,ti	33305
#14	cycling:ab,ti	69296
#13	'strength train*':ab,ti	6758
#12	'cardio fitness':ab,ti	14
#11	'cardio train*':ab,ti	21
#10	jogging:ab,ti	2181
#9	running:ab,ti	77445
#8	pilate*:ab,ti	890
#7	yoga:ab,ti	7291
#6	aerobic*:ab,ti	109708
#5	'resistance train*':ab,ti	10434
#4	sport*:ab,ti	105531
#3	walk*:ab,ti	172985
#2	exercis*:ab,ti	412241
#1	'physical* activ*':ab,ti	165064

Limiters: Human, English and Embase database only (not Medline)
:ab – abstract, :ti – title /de – index term /exp – exploded index term

CoCHRANE (CENTRAL)

460

ID	Search
#1	“Physical Activity”:ti,ab
#2	exercise:ti,ab
#3	walk*:ti,ab
#4	sport*:ti,ab
#5	"resistance training":ti,ab
#6	aerobic:ti,ab
#7	yoga:ti,ab
#8	pilate*:ti,ab
#9	running:ti,ab
#10	jogging:ti,ab
#11	"cardio training":ti,ab
#12	"cardio fitness":ti,ab
#13	"strength training":ti,ab
#14	cycling:ti,ab
#15	bicycling:ti,ab
#16	"active transport":ti,ab
#17	"endurance training":ti,ab
#18	MH "physical Activity"
#19	#1 OR #2 OR #3 OR #4 OR #5 OR #6 OR #7 OR #8 OR #9 OR #10 OR #11 OR #12 OR #13 OR #14 OR #15 OR #16 OR #17 OR #18
#20	"type 2 diabetes":ti,ab
#21	"type II diabetes":ti,ab
#22	NIDDM:ti,ab

#23	T2D:ti,ab
#24	T2DM:ti,ab
#25	DM2:ti,ab
#26	"maturity onset diabetes":ti,ab
#27	"slow onset diabetes":ti,ab
#28	"non-insulin dependent":ti,ab
#29	"adult diabetes":ti,ab
#30	"late-onset diabetes":ti,ab
#31	"stable diabetes":ti,ab
#32	MH diabetes
#33	#20 OR #21 OR #22 OR #23 OR #24 OR #25 OR #26 OR #27 OR #28 OR #29 OR #30 OR #31 OR #32
#34	"metabolic syndrome":ti,ab
#35	"metabolic X":ti,ab
#36	"syndrome X":ti,ab
#37	cardiometabolic:ti,ab
#38	"cardiovascular risk factors":ti,ab
#39	"metabolic cardiovascular":ti,ab
#40	"cardiovascular syndrome":ti,ab
#41	"blood pressure":ti,ab
#42	triglycerides:ti,ab
#43	cholesterol:ti,ab
#44	"high-density lipoprotein ratio":ti,ab
#45	"high -density lipoprotein":ti,ab
#46	"low-density lipoprotein":ti,ab
#47	"glycated haemoglobin":ti,ab
#48	"glycated hemoglobin":ti,ab
#49	"glycosylated haemoglobin":ti,ab
#50	"waist circumference":ti,ab
#51	"waist-to-hip ratio":ti,ab
#52	MH "metabolic syndrome"

#53 #34 OR #35 OR #36 OR #37 OR #38 OR #39 OR #40 OR #41 OR #42 OR #43 OR #44 OR #45 OR #46 OR #47 OR #48 OR #49 OR #50 OR #51 OR #52
#54 "randomized controlled trial":ti,ab
#55 "randomised controlled trial":ti,ab
#56 "pragmatic clinical trial":ti,ab
#57 "equivalence trial":ti,ab
#58 RCT:ti,ab
#59 MH "randomized controlled trial"
#60 #54 OR #55 OR #56 OR #57 OR #58 OR #59
#61 #19 AND #33 AND #53 AND #60

S2. Forest plot for studies involving aerobic exercises

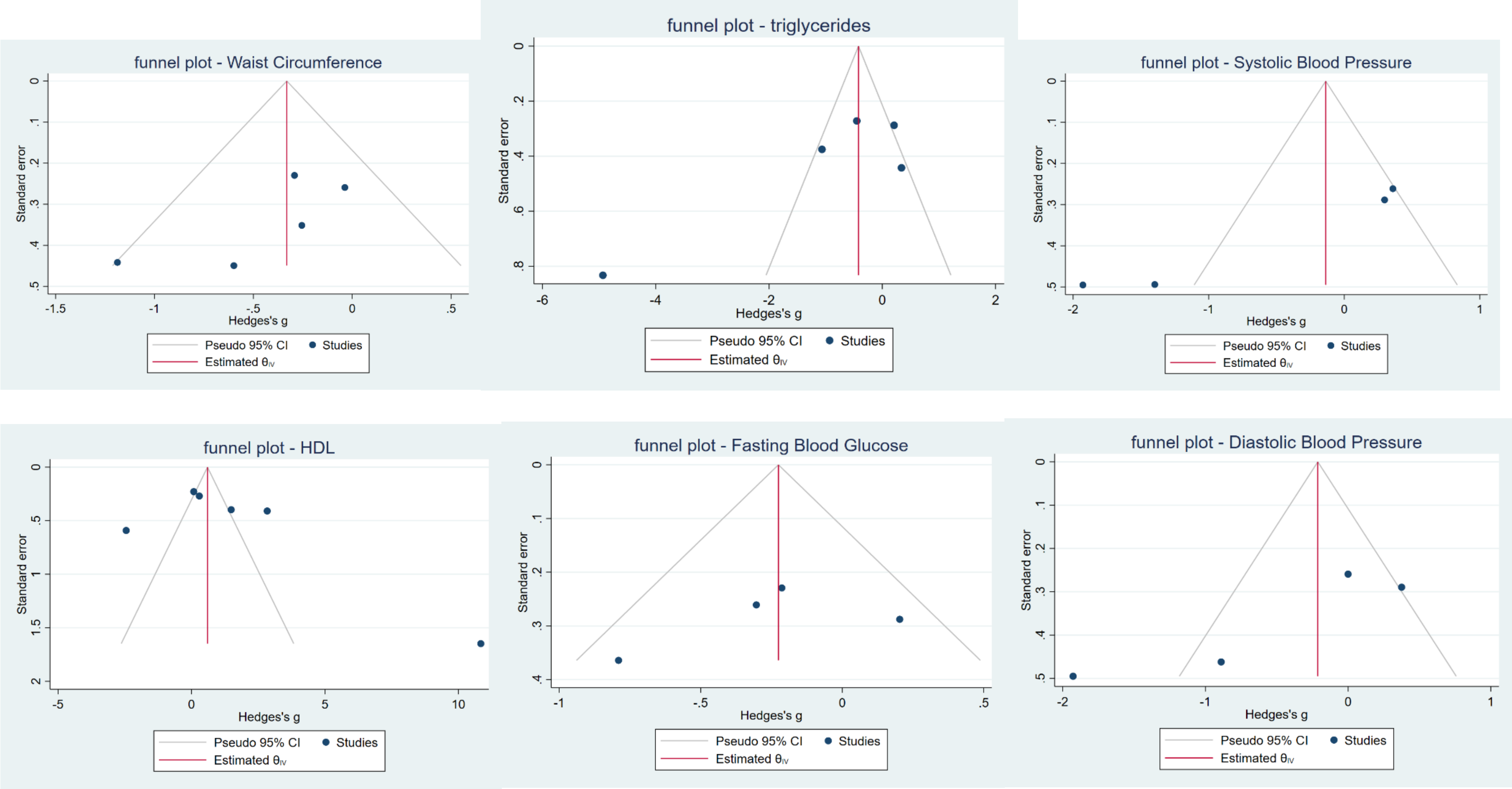

θ is the odds of a positive outcome under the treatment

S3. Forest plot for studies involving resistance exercises

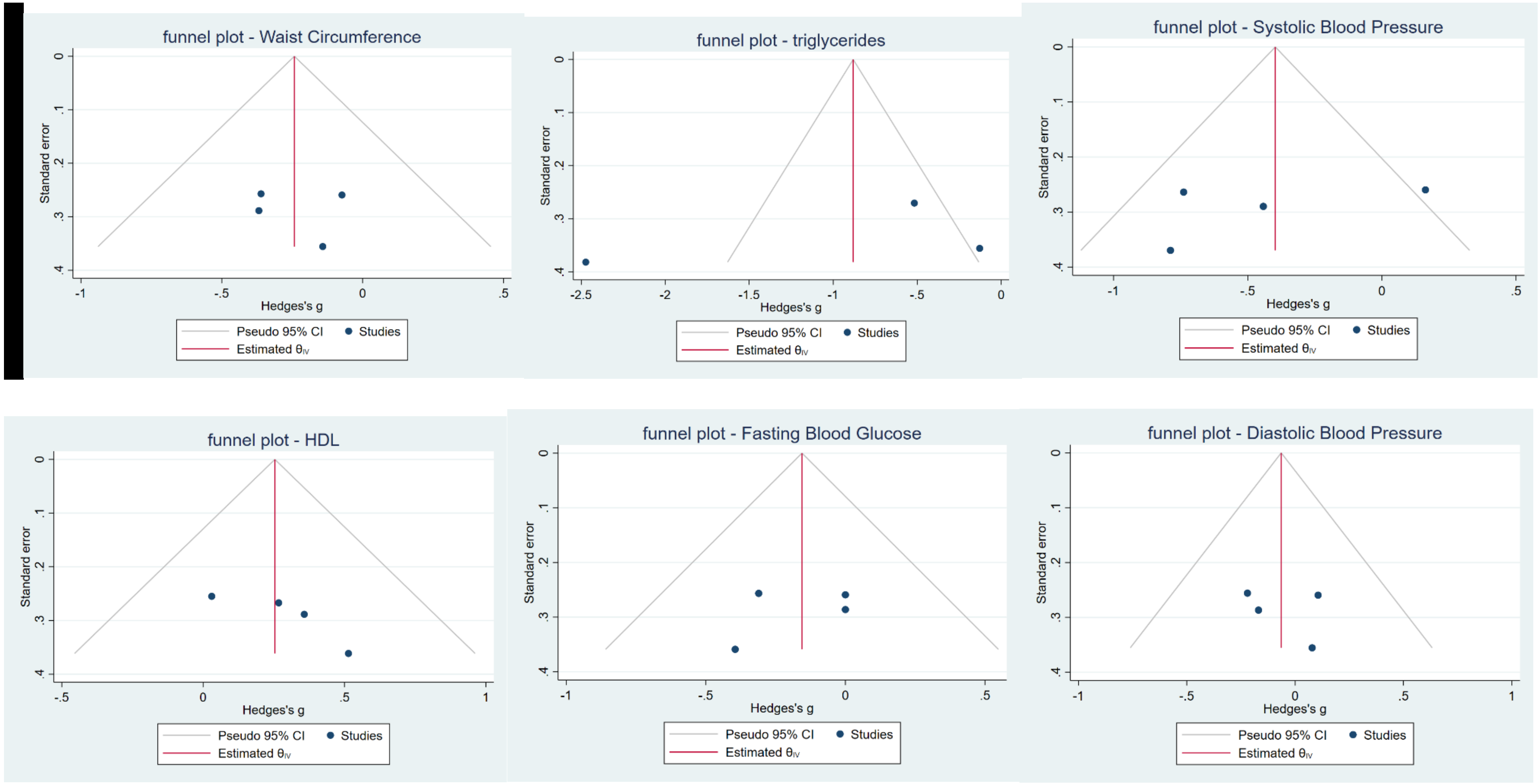

θ is the odds of a positive outcome under the treatment

S4: Effect of aerobic exercise on additional MetS markers in T2DM

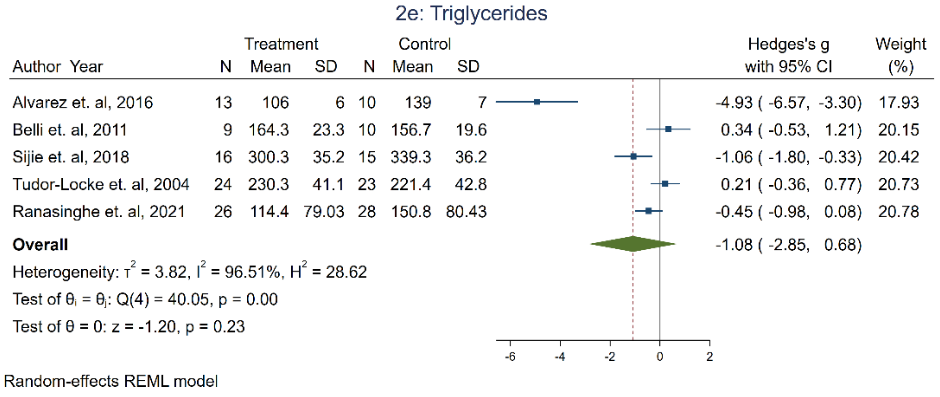

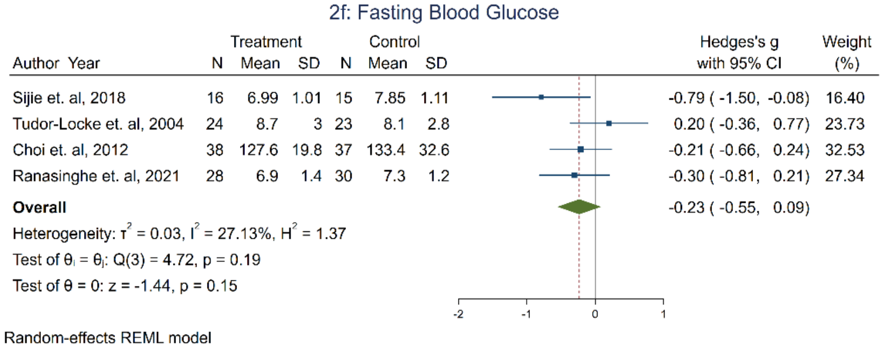

Effect of aerobic exercise on MetS markers in T2DM. Data are reported as Hedge's G (effect size) and 95% confidence interval (CI). The diamond at the bottom presents the overall effect. The plotted squares denote effect sizes, and the whiskers denote their 95% CIs [2, 5, 22, 23, 7, 18].

S5: Effect of resistance exercise on MetS markers in T2DM

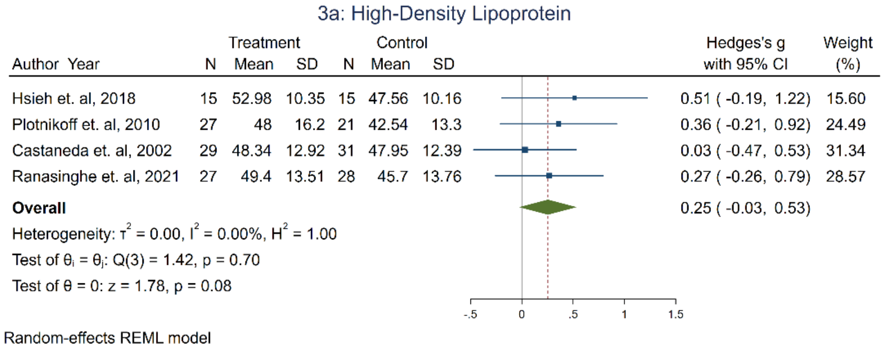

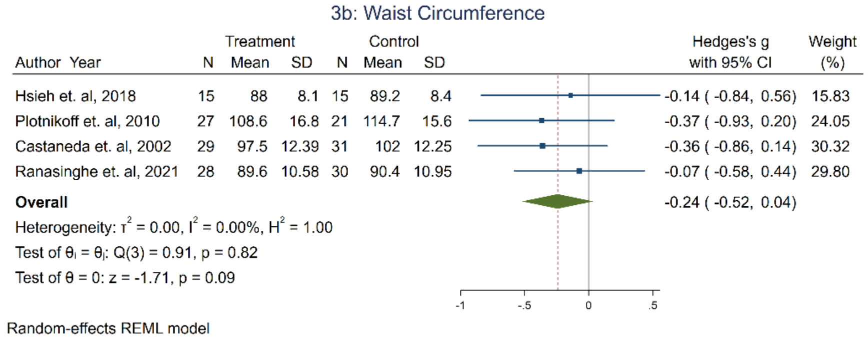

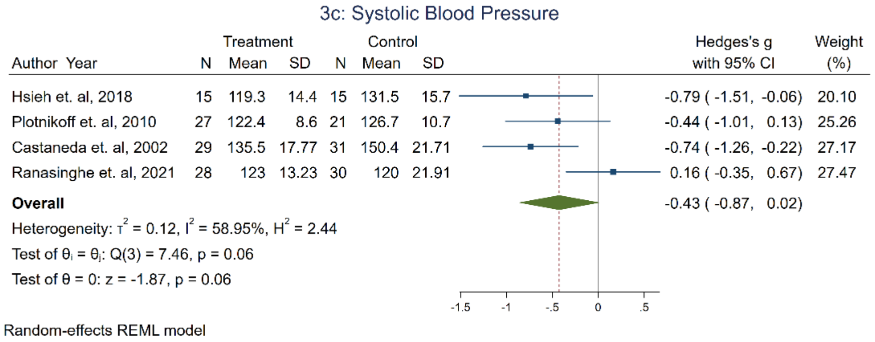

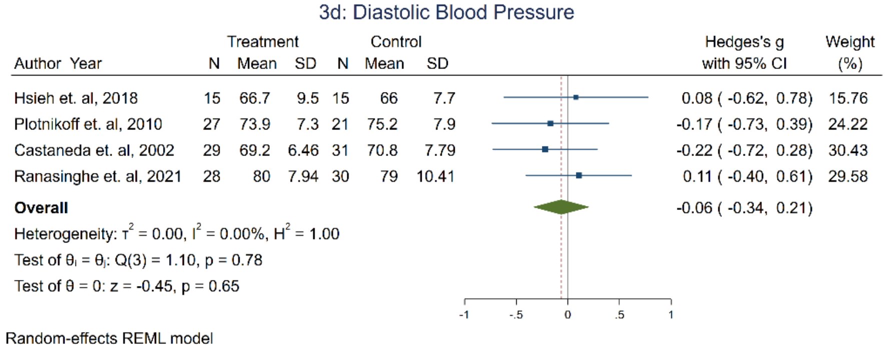

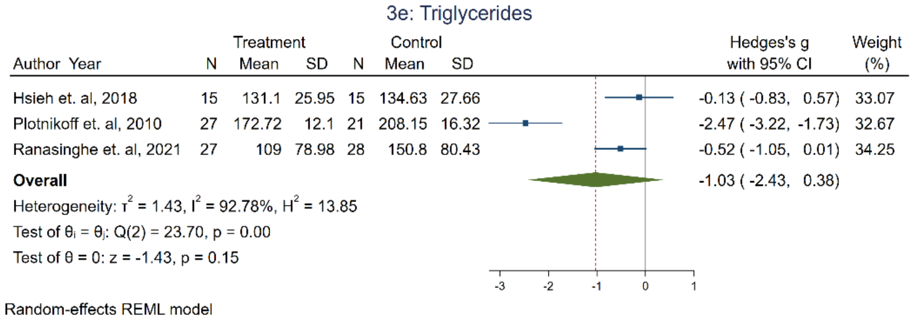

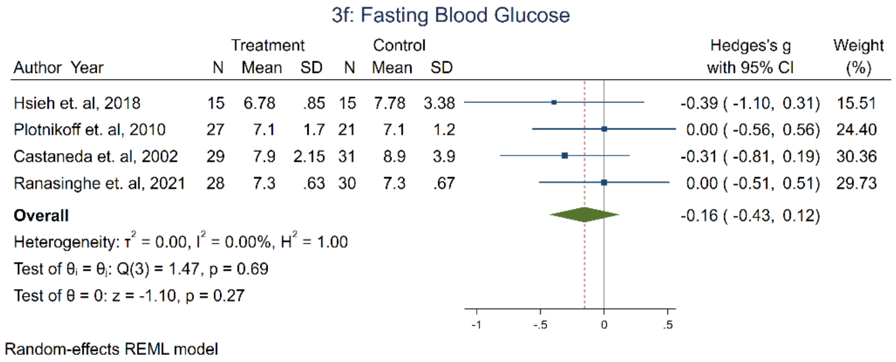

Effect of resistance exercise on MetS markers in T2DM. Data are reported as Hedge's G (effect size) and 95% confidence interval (CI). The diamond at the bottom presents the overall effect. The plotted squares denote effect sizes, and the whiskers denote their 95% CIs [13], [17], [18], [6].

S6: characteristics of studies included in the review

Study author, year	Study details (aim, design, setting, country)	Sample characteristics (population, sample size)	PA program description	Endpoints
Argurs-Collins et. al, 1997	To evaluate a weight loss and exercise program designed to improve T2DM management in older African Americans.<br>RCT, parallel group.<br>Clinical/community setting.<br>USA.	African American men and women aged 55-59 years and with T2DM.<br>N = 64 (IG = 32, CG = 32)	5-min warm-up, 20-min low-impact aerobic activity, and 5-min cool-down exercises.<br>30 mins per session, at least 3x per week for 6 months.<br>Moderate intensity.	BMI ($p < 0.05$) *<br>HbA1c ($p < 0.05$) *<br>DBP ($p < 0.05$) *<br><br>WHR<br>DBP, TC, HDL, LDL, TG.
Alvarez et. al, 2016	To investigate the effects of low-volume, high intensity interval training on cardiometabolic risk and exercise capacity in women with T2DM.<br>RCT, parallel group.<br>Clinical setting.<br>Chile.	Adult overweight or obese (BMI between 25 and 35kg/m2) adult women with established diagnosis of T2DM for at least 12 months<br>N = 23 (IG = 13, CG = 10)	Participants jog/run and walking.<br>3x per week for 16 weeks.<br>Low and high-intensity exercise.	FBG ($p < 0.05$) *<br>HbA1c ($p < 0.05$) *<br>SBP ($p < 0.05$) *<br>TG ($p < 0.05$) *<br>BMI ($p < 0.05$) *<br>WC ($p < 0.05$) *<br><br>DBP, LDL, TC, HDL
Balducci et. al, 2010	To assess the efficacy of an intensive exercise intervention strategy in improving PA, HbA1c, and other cardiovascular risk factors in patients with T2DM.<br>RCT, parallel group.<br>Clinical setting.<br>Italy.	Patients with T2DM International Diabetes Federation (IDF) criteria for metabolic syndrome.<br>N = 606 (IG = 303, CG = 303).	Two supervised sessions of progressive mixed (aerobic and resistance) training.<br>150 minutes per week for 12 months.<br>Moderate intensity.	HbA1c ($p < 0.05$) *<br>SBP ($p < 0.05$) *<br>DBP ($p < 0.05$) *<br>HDL ($p < 0.05$) *<br>LDL ($p < 0.05$) *<br>WC ($p < 0.05$) *<br><br>BMI, TC, TG

Bassi et. al, 2016	To investigate the impact of concurrent aerobic and resistance training programs on metabolic profile, glycaemic control, and exercise capacity in patients with T2DM.<br>RCT, parallel group.<br>Laboratory setting.<br>Brazil.	patients with T2DM and being sedentary for at least 6 months<br>N = 41 (IG = 21, CG = 20)	Supervised sessions including 5-min warm-up, 60-min CART training, and 5-min cool down.<br>Session lasted 1 hour 10 mins for 12 weeks<br>Moderate intensity<br>combined	HbA1c ($p < 0.005$) *<br>TC ($p < 0.005$) *<br>SBP (p < 0.005) *<br>WC ($p < 0.005$) *<br><br>BMI, LDL, HDL, DBP
Belli et. al, 2011	To analyse the effects of overground walking training at ventilatory threshold velocity on glycaemic control, body composition, physical fitness, and lipid profile in women with T2DM.<br>RCT, parallel group.<br>Clinical setting.<br>Brazil.	Housewives who are non-smokers and inactive, and have T2DM.<br>N = 41 (IG = 21, CG = 20).	Supervised walking exercise including stretching exercise.<br>20-60 mins per session for 12 weeks.<br>Moderate intensity.	HbA1c ($p < 0.005$) *<br>BMI ($p < 0.005$) *<br><br>FBG, SBP, DBP, TC, HDL, LDL, TG, WC
Castaneda et. al, 2002	To determine the efficacy of high-intensity progressive resistance training (PRT) on glycaemic control in older adults with T2DM.<br>RCT, parallel group.<br>Clinical setting.<br>USA.	Community-dwelling Latinos >55 years of age and have T2DM of at least 3 years duration.<br>N = 62 (IG = 31, CG = 31).	Supervised session consisting of 5-min warm-up, 35-min PRT, and 5-min cool-down<br>45 mins per session, 3x per week for 16 weeks.<br>High intensity, resistance training.	HbA1c ($p <0.001$) *<br>SBP ($p <0.001$) *<br><br>FBG, DBP, HDL, LDL,<br>WC

Choi et. al, 2012	To examine the effects of exercise on sRAGE and its association with diverse cardiovascular risk factors and indicators of atherosclerosis in patients with T2DM.<br>RCT, parallel group.<br>Home setting.<br>Korea.	Women with T2DM with a stable body weight and sedentary.<br>N = 75 (IG = 38, CG = 37)	Unsupervised walking at moderate exercise capacity.<br>60 mins per session, 5x per week for 12 weeks.<br>Moderate intensity	SBP ($p < 0.05$) *<br>WC ($p < 0.001$) *<br>HbA1c ($p < 0.05$) *<br>FBG ($p < 0.05$) *<br><br>TC, TG, HDL, LDL
Church et. al, 2010	To examine the effects of aerobic training alone, resistance training alone, and a combination of both on Hb1Ac in individuals with T2DM.<br>RCT, parallel group. Clinical setting.<br>USA.	Sedentary 30- to 75-year-old adults with T2DM and HbA1c levels of 6.5% to 11.0%.<br>N = 262 (RT = 73, AT = 72, CT = 76, CG = 41).	Resistance training, aerobic training, and combined exercise consisting of aerobic and resistance exercises.<br>150 mins/ week, 3 days per week for 9 months.<br>Moderate intensity.	HbA1c ($p < 0.05$) *<br>WC ($p < 0.05$) *<br>FBG ($p < 0.05$) *<br><br>BMI, LDL,<br>HDL, SBP, DBP, TG
Conners et. al, 2017	To determine if an underwater treadmill walking program featuring gradual and progressive increases in walking speed and duration has a positive effect on glycaemic control, metabolic health, cardiovascular function, body composition, and leg strength in individuals with T2DM.<br>RCT, parallel group.<br>Laboratory setting.<br>USA.	Sedentary middle-aged adults with diagnosis of type 2 diabetes for a minimum of 2 years has a sedentary lifestyle.<br>N = 26 (IG = 13, CG = 13).	12-week program of underwater treadmill walking<br>15 mins, 3x per week for 12 weeks<br>CG continued their usual treatment	HbA1c ($p < 0.05$) *<br>WC ($p < 0.05$) *<br>HDL ($p < 0.05$) *<br>LDL ($p < 0.05$) *<br><br>TG, DBP, SBP, TC

Emerenziani et. al, 2015	To evaluate the effects of aerobic training intervention, based on heart rate at aerobic gas exchange threshold, on clinical and physiological parameters in obese elderly subjects with type 2 diabetes.<br>RCT, parallel group.<br>Clinical setting.<br>Italy.	Obese elderly subjects with T2DM<br>N = 30 (IG = 15, CG = 15).	Aerobic training consisting of 5 mins warm-up, 30 mins aerobic training, and 5 mins cool down. CG had no exercise intervention.<br>Each session lasted 50 mins, 2x per month for 3-month.<br>Moderate intensity.	HbA1c ($p < 0.05$) *<br><br>BMI, HDL, LDL, TC, BMI
Gordon et. al, 2008	To investigate the effect of Hatha yoga and conventional physical training exercise intervention on clinical and biochemical parameters in patients with type 2 diabetes.<br>RCT, parallel group.<br>Clinical setting.<br>Jamaica.	People with type 2 diabetes mellitus, 40-70 years old<br>N = 231 (IG1 = 77, IG2 = 77, CG = 77)	Aerobic exercise consisting of 15 mins of warm-up exercises, 30 mins of aerobic walking, 20 mins of body flexibility exercises, 20 mins of aerobic dance, 25 mins of games and 10 mins of warm-down exercises. CG had no exercise intervention.<br>3-4 times per week for 6 months	FBG ($p < 0.05$) *<br>HbA1c ($p < 0.05$) *<br>SBP ($p < 0.05$) *<br>DBP ($p < 0.05$) *<br><br>LDL, HDL
Hangping et. al, 2019	To evaluate the effects of a novel, low-volume, high-intensity Progressive Resistance Training (PRT) technique on blood glucose control in elderly Chinese patients with T2DM.<br>RCT, parallel group.<br>Clinical setting.<br>China.	People with diagnosis of type 2 Diabetes, aged 50 to 75 years.<br>N = 265 (IG = 165, CG = 100).	Supervised progressive resistance training. CG had no exercise intervention.<br>5–10 mins weekly.<br>High intensity.<br>6 months.	HDL ($p < 0.05$) *<br>HbA1c ($p < 0.05$) *<br>LDL ($p < 0.05$) *<br>TC ($p < 0.05$) *<br><br>FBG, TG, SBP, DBP
Hsieh et. al, 2018	To investigate the effects of 12 weeks of resistance training on muscle function, physical performance, cardiometabolic risks, and QoL in older people with T2DM.	Participants aged 65 to 80 years with a diagnosis of T2DM.<br>N = 30 (IG = 15, CG = 15).	Supervised resistance exercise. CG had no exercise intervention.<br>3 times per week for 12 weeks.<br>Moderate intensity.	SBP ($p < 0.05$) *<br>WC ($p < 0.05$) *<br>FBG ($p < 0.05$) *<br>DBP ($p < 0.05$) *<br>HbA1c ($p < 0.05$) *

	RCT, parallel group.<br>Clinical setting.<br>Taiwan.			BMI, HDL, LDL, TG, TC
Huimin et. al, 2014	To investigate the effects of aerobic exercise program on glucose control and BP in people with T2DM<br>RCT, parallel group<br>Clinical setting<br>Mozambique	Participants aged between 40 and 70, diagnosed with type 2 diabetes<br>N = 41 (IG = 31, CG = 10)	Supervised aerobic exercises. CG had no exercise intervention.<br>45 min/session, 3–5times/week.<br>Moderate intensity<br>12 weeks	FBG ($p < 0.05$) *<br>SBP ($p < 0.05$) *<br>DBP ($p < 0.05$) *<br><br>WC, BMI, HbA1c
Lam et. al, 2008	To assess the effect of tai chi on glycated haemoglobin, blood pressure and health status in adults with T2DM<br>RCT, parallel group<br>Community setting<br>Australia	Participants who were 30 years or over, and had a diagnosis of type 2 diabetes<br>N = 46 (IG = 24, CG = 22)	Tai Chi. CG had no exercise intervention<br>Two sessions per week for 3 months, then once per week for 3 months<br>moderate intensity	SBP, DBP, HbA1c, TC, TG, BMI
Lambers et. al, 2008	To investigate the influence of combined exercise training on indices of obesity, diabetes, and cardiovascular risk in adults with T2DM<br>RCT, parallel group<br>Clinical setting<br>Belgium	Participants who were 30 years or over, and had a diagnosis of type 2 diabetes<br>N = 46 (IG1 = 17, IG2 = 18, CG = 11)	Supervised combined exercise training including stretching, circuit training: walking, cycling, and stepping.<br>60 mins 3x per week, for 3 months<br>CG continued normal activities, without exercise	HbA1c ($p < 0.05$) *<br>TC ($p < 0.05$) *<br>HDL ($p < 0.05$) *<br><br>LDL, TG, WC
Plotnikoff et. al, 2010	To investigate whether a home-based resistance training (RT) program could provide benefits to obese patients with type 2 diabetes	Participants who were obese and sedentary, and had a diagnosis of type 2 diabetes<br>N = 48 (IG = 27, CG = 21)	Supervised prescribed resistance training<br>3 non-consecutive days per week, for 16-weeks.<br>Moderate intensity	HDL ($p < 0.05$) *<br><br>BMI, HbA1c, LDL, TG, TC, WC,

	RCT, parallel group<br>Home-based setting<br>Canada		CG had no exercise intervention	SBP, DBP, WHR, FBG
Ranasinghe et. al, 2021	To examine the effects of aerobic training and resistance training compared to standard care on glycaemic control in south Asian Sri Lankan adults with type 2 diabetes<br>RCT, parallel group<br>Clinical setting<br>Sri Lanka	Adults aged 35e65 years and diagnosed with T2DM<br>N = 48 (IG = 27, CG = 21)	Supervised aerobic training and resistance training<br>75-min per session, 2 times per week for 12 weeks.<br>Moderate intensity<br>CG had no exercise intervention	FBG ($p < 0.05$) *<br>HbA1c ($p < 0.05$) *<br>TC ($p < 0.05$) *<br>HDL ($p < 0.05$) *<br><br>LDL, TG, SBP, DBP, BMI, WC
Shantakumari et. al, 2013	To assess the effectiveness of yoga in the management of dyslipidaemia in patients with type 2 diabetes<br>RCT, parallel group<br>Clinical setting<br>India	Adults with type 2 diabetes and dyslipidaemia<br>N = 100 (IG = 50, CG = 50)	Yoga postures<br>30-35 min daily for 3 months<br>CG continued with their oral medications without yoga	WHR ($p < 0.05$) *<br>TC ($p < 0.05$) *<br>TG ($p < 0.05$) *<br>LDL ($p < 0.05$) *<br><br>HDL, FBG
Shenoy et. al, 2009	To evaluate the effects of progressive resistance training and aerobic exercise on glycaemic control, blood pressure, heart rate, muscle strength and control of T2DM<br>RCT, parallel group<br>Clinical setting<br>India	Inactive adults with type 2 diabetes<br>N = 20 (IG = 10, CG = 10)	Progressive resistance training, aerobics, and control group without any exercise intervention.<br>2x per week for 16 weeks<br>Moderate-high intensity	HbA1c ($p < 0.05$) *<br>FBG ($p < 0.05$) *<br>SBP ($p < 0.05$) *<br>DBP ($p < 0.05$) *<br><br>BMI, WC

Sigal et. al, 2007	To determine the effects of aerobic training alone, resistance training alone and combined exercise training on glycated haemoglobin in patients with T2DM<br>RCT, parallel group<br>Community setting<br>Canada	People with type 2 diabetes and has HbA1c between values of 6.6%-99.9%<br>N = 251 (IG1 = 60, IG2 = 64, IG3 = 64, CG = 63)	Supervised aerobic training on treadmills or bicycle ergometers. The resistance training group performed 7 different exercises on weight machines. The combined exercise training group did the full aerobic training program plus the full resistance training program. CG had no exercise intervention.<br>3x per week, for 6 months	HbA1c ($p < 0.05$) *<br><br>BMI, WC, SBP, DBP, HDL, LDL, TG, TC
Sijie et. al, 2018	To investigate the pleiotropic effects of supervised exercise training at maximal fat oxidation in tensity on body composition, lipid profile, glycaemic control, insulin sensitivity and serum adipokine levels in older women with T2DM<br>RCT, parallel group<br>Laboratory setting<br>China	Women with type 2 diabetes, aged 60-69 years<br>N = 32 (IG = 16, CG = 16)	FATmax exercise training involving stretches, 20-40 min of walking or running<br>20-60 min per day, 3 times per week for 12 weeks<br>High intensity	BMI ($p < 0.05$) *<br>WC ($p < 0.05$) *<br>WHR ($p < 0.05$) *<br>FBG ($p < 0.05$) *<br>LDL ($p < 0.05$) *<br>TG ($p < 0.05$) *<br>TC ($p < 0.05$) *<br><br>HDL, SBP, DBP
Tudor-Locke et. al, 2004	To assess the impact of a PA intervention for people with T2DM<br>RCT, parallel group<br>community setting<br>Canada	People with type 2 diabetes, aged 40-60 years<br>N = 47 (IG = 24, CG = 23)	Use of pedometers to measure steps per day<br>16 weeks<br>Not specified	WC ($p < 0.05$) *<br><br>FBG, HDL, LDL, TG, TC, SBP, DBP

Vancea et. al, 2009	To compare the influence of guided and structured physical exercise program on glycaemic control and body composition in people with T2DM.<br>RCT, parallel group<br>clinical setting<br>Brazil	People with type 2 diabetes, aged 40-65 years<br>N = 47 (IG1 = 14, IG2 = 9, CG = 17)	Exercise group 1 did 3 workout sessions per week consisting of warm-up (5 mins): stretching exercises; 2) main exercises (30 mins): walking on the treadmill, and 3) back to relaxation (10 mins). Group 2 repeated same exercise but had it for 5 workout sessions per week<br>20 weeks	BMI ($p < 0.05$) *<br>FBG ($p < 0.05$) *<br><br>DBP, SBP, HbA1c, HDL, LDL, WC
Wang et. al, 2019	To compare the effects of aerobic exercise alone and resistance training alone as well as the combination of both on glycaemic control in people with T2DM.<br>RCT, parallel group<br>Community setting<br>China	People with type 2 diabetes, aged 18-85 years<br>N = 794 (IG1 = 200, IG2 = 199, IG3 = 198, CG = 197)	Group 1 had health literacy intervention. Group 2 walked 30-70 min, 3 to5 days a week. Group 3 had both literacy and walking intervention. CG had no exercise intervention.<br>24 months	LDL ($p < 0.05$) *<br><br>HDL, SBP, DBP, WC, HbA1c
Yavari et. al, 2012	To evaluate the effectiveness of health literacy and exercise-focused interventions on clinical outcomes among chines patients with T2DM.<br>RCT, parallel group<br>Clinical setting<br>Iran	People with type 2 diabetes, and were inactive<br>N = 60 (IG1 = 15, IG2 = 15, IG3 = 15, CG = 15)	Group 1 performed resistance exercise; Group 2 performed aerobics Group 3 did the aerobic exercise plus resistance training. CG had no exercise intervention<br>52 weeks<br>20-60 mins per session, 3 times a week for 12 months.	HbA1c ($p < 0.05$) *<br>FBG ($p < 0.05$) *<br>SBP ($p < 0.05$) *<br>DBP ($p < 0.05$) *<br>TG ($p < 0.05$) *<br><br>WC, HDL, LDL

* Significant difference between intervention group and control group following exercise intervention, FBG fasting blood glucose, SBP systolic blood pressure, DBP diastolic blood pressure, WC waist circumference, TG triglycerides, TC total cholesterol, WHR waist-to-hip ratio, HbA1c glycated haemoglobin, BMI body mass media, HDL high density lipoprotein, LDL low density lipoprotein, BMI body mass index, IG intervention group, CG control group.
